# Chimeric antigen receptor macrophage therapy for breast tumours mediated by targeting the tumour extracellular matrix

**DOI:** 10.1038/s41416-019-0578-3

**Published:** 2019-10-01

**Authors:** Wenlong Zhang, Ling Liu, HuiFang Su, Qin Liu, Jie Shen, Hanren Dai, Wei Zheng, Yan Lu, Weijie Zhang, Yuncheng Bei, Pingping Shen

**Affiliations:** 1 State Key Laboratory of Pharmaceutical Biotechnology and The Comprehensive Cancer Center, Nanjing Drum Tower Hospital, MOE Key Laboratory of Model Animal for Disease Study, School of Life Sciences, Nanjing University, 210046 Nanjing, PR China; 2 The Comprehensive Cancer Center, Nanjing Drum Tower Hospital, The Affiliated Hospital of Nanjing University Medical School, 210008 Nanjing, PR China; 3 Department of General Surgery, Drum Tower Hospital, Medical School of Nanjing University, 210008 Nanjing, Jiangsu China; 4 College of Life Sciences, Peking University, 100871 Beijing, PR China

**Keywords:** Breast cancer, Cell delivery, Immunization

## Abstract

**Background:**

The extracellular matrix (ECM) is essential for malignant tumour progression, as it is a physical barrier to various kinds of anticancer therapies. Matrix metalloproteinase (MMPs) can degrade almost all ECM components, and macrophages are an important source of MMPs. Studies using macrophages to treat tumours have shown that macrophages can enter tumour tissue to play a regulatory role.

**Methods:**

We modified macrophages with a designed chimeric antigen receptor (CAR), which could be activated after recognition of the tumour antigen HER2 to trigger the internal signalling of CD147 and increase the expression of MMPs.

**Results:**

Although CAR-147 macrophage treatment did not affect tumour cell growth in vitro compared with control treatment. However, we found that the infusion of CAR-147 macrophages significantly inhibited HER2-4T1 tumour growth in BALB/c mice. Further investigation showed that CAR-147 macrophages could reduce tumour collagen deposition and promote T-cell infiltration into tumours, which were consistent with expectations. Interestingly, the levels of the inflammatory cytokines TNF-α and IL-6, which are key factors in cytokine release syndrome, were significantly decreased in the peripheral blood in CAR-147 macrophage-transfused mice.

**Conclusion:**

Our data suggest that targeting the ECM by engineered macrophages would be an effective treatment strategy for solid tumours.

## Background

Cancer immunotherapy aims to promote or modify immune cells (especially T cells) to attack cancer cells while keeping normal cells intact. The innate and adaptive immune systems play vital roles in the immune surveillance, identification and destruction of cancer cells.^1,2^ Adoptive cellular therapy ranked first in the top ten scientific and technological advances in 2013. Recently, adoptive cellular therapy based on DCs, T cells, NK cells, etc. has achieved good effects on tumours. Among these cells, chimeric antigen receptor (CAR)-modified T cells (CAR-T cells) have developed very rapidly in recent years. This concept was first proposed in 1989.^3^ In recent years, the success of CAR-T cell immunotherapy targeting the B-cell lineage differentiation antigen CD19 in B-cell malignancies has provided new opportunities for the treatment of cancer. Although the therapeutic effect of CAR-T cells on haematological malignancies is impressive, the results of treating solid tumours with CAR-T cells have been less than ideal.^4^

Whether CAR-T cells can reach the tumour site is a prerequisite for their anti-tumour effect. When T cells extravasate from blood vessels, they need to pass through dense tumour tissue to reach the target cell location. Physical barriers formed by the stroma characterise many types of cancer, and the resulting high tissue pressure further prevents the extravasation of T cells. The tumour-associated extracellular matrix (ECM) and fibroblasts have immunomodulatory effects.^5,6^ Compared with wild-type mice, mice with deficient expression of the ECM protein tenascin have higher effector immune cell infiltration into tumours.^7^ Tumour tissue contains abundant and special ECM such as collagen and proteoglycan. The dense tissue morphology forms a physical barrier that limits the free migration of T cells.^8^ Some studies have shown that a high blood vessel density is associated with high T-cell and B-cell abundances in tissue sections from patients with solid tumours.^9^ Some successes have been achieved in animal models by utilising fibroblast activation protein (FAP) CAR-T cells to reduce the number of tumour fibroblasts to counteract these physical barriers.^10^ Heparanase, an enzyme that degrades the matrix, can promote CAR-T cell infiltration and anti-tumour efficacy in solid tumours.^11^

The ECM is generated by the highly organised interactions of fibre molecules, proteoglycans, glycoproteins, glycosaminoglycans and other macromolecules, including approximately 300 different proteins.^12^ Its synthesis and degradation are mainly regulated by matrix metalloproteinase (MMPs) and tissue inhibitors of metalloproteinases (TIMPs). MMPs are a family of calcium and zinc-dependent proteolytic enzymes that currently includes at least 26 subtypes that degrade almost all ECM and basement membrane components.^13^ TIMPs are an important family of enzymes that regulate the activity of MMPs, inhibiting the activity of MMPs and reducing the degradation of the ECM. Four members of the TIMP family have been found: TIMP-1, TIMP-2, TIMP-3, and TIMP-4. TIMPs form TIMP−MMP complexes with MMPs at a ratio of 1:1, thereby blocking the binding of MMPs to substrates and inhibiting the activity of MMPs.^14^ The overall proteolytic activity is determined by the ratio of MMP−TIMP, which in turn affects the deposition and degradation of the ECM.^15^

Macrophages are an important source of MMPs.^16^ Kupffer cells (KCs) can express a variety of MMPs, such as MMP-9, MMP-12 and MMP-13, to degrade the matrix, which is beneficial in the repair of liver damage and liver fibrosis.^17,18^ Some studies have shown that the infusion of bone marrow-derived macrophages in mice can significantly alleviate liver fibrosis and improve liver function.^19^ In a mouse model of acute liver injury induced by acetaminophen, the transplantation of KCs can also protect liver cells and reduce liver damage.^20^ Clinically, pulmonary macrophage transplantation is an effective cellular therapy in children with pulmonary alveolar proteinosis.^21–23^ These clinical data also suggest that macrophage transplantation is safe and well tolerated.

Numerous studies have shown that macrophages from various sources can coexist in tumours. Locally self-maintained macrophages are a part of the TAM population, but macrophages recruited from the peripheral blood account for the majority of TAMs,^24^ which suggests that injected macrophages can infiltrate tumours. Hence, to prevent the matrix in the tumour from reducing the effect of anticancer drugs, hindering the entry of T cells, and promoting tumour growth, we designed a chimeric antigen receptor targeting HER2 for macrophages, with the hope of activating MMPs to degrade the matrix and broaden the path for T cells entry into the tumour. HER2 is a well-established therapeutic target in breast cancer. The 4T1 murine breast tumour model, which show similarities to the human disease, was used to study the effectiveness of CAR-147 macrophages in vivo.

## Methods

### Cells

4T1 cells and Raw264.7 cells were purchased from Shanghai Institutes for Biological Sciences, Chinese Academy of Sciences. 4T1 cells were cultured in RPMI1640, Raw264.7 cells were cultured in DMEM. All media supplemented with 10% FBS (Gibco), 2 mM l-glutamine, 100 units/ml penicillin and 100 units/ml streptomycin. The cells were kept in a humidified atmosphere of 5% CO_2_ at 37 °C.

### Reagents

PE-conjugated anti-mouse CD206 (Clone M1), PE-conjugated anti-mouse MHCII, PE-conjugated anti-mouse PDL1, PerCP-conjugated anti-mouse CD40, PE-Cy7-conjugated anti-mouse CD86, PE-conjugated anti-mouse CD80 and corresponding isotype controls were purchased from BD Pharmingen (San Diego, CA). Penicillin and streptomycin, DCFH-DA probe, HRP-conjugated goat anti-mouse IgG (H + L) are from Beytotime (Haimen, Jiangsu, China). Annexin V/7-AAD apoptosis kit was purchased from BD Pharmingen (San Diego, CA). Alexa Fluor® 647 AffiniPure F(ab′)_2_ Fragment Rabbit Anti-Mouse IgG (H + L) was purchased from Jackson Immunoresearch (West Grove, PA, USA). Matrigel was purchased from Sigma (St. Louis, MO).

### Mice

Nine-week-old female BALB/c mice and nude mice were purchased from Nanjing Biomedical Research Institute of the Nanjing University, Nanjing, China and bred in our animal facilities under specific pathogen-free conditions. All experiments related to animals were approved by the Institutional Animal Care and Use Committee, Nanjing University. The average weight of mice at the start of the experiment was 20 g. Vendor health reports indicated that the mice were free of known viral, bacterial and parasitic pathogens. Animals were housed in an SPF facility, using five mice per cage with sterile wood shavings as bedding. Animal welfare was assessed daily by the authors. All animals were either treated (where possible) or humanely euthanized at any sign of illness or stress.

### Macrophage/tumour cell coculture

Raw264.7 cells were cocultured with 4T1 cells in a cell−cell contact fashion for 24 h or 48 h. After coculture, the 4T1 cells and Raw264.7 cells were harvested, layered in a 40 and 70% Percoll gradient (Sigma-Aldrich, St Louis, MO), and centrifuged at 3000 rpm for 30 min without brake engagement. The Raw264.7 cells at the interface were collected. All the coculture experiments in this study were performed with cell−cell contact.

### Tumour cell invasion assay

4T1 tumour cells (5 × 10^5^ cells/ml) were placed in Matrigel-coated invasive chamber (24 wells, 8 mm pore size); after 24 h, the 4T1 tumour cells invading the other side of the chamber were fixed and stained with crystal violet.

### Phagocytosis

Phagocytosis assays were performed by fluorescent red latex beads (1 μM diameter, L-2778, Sigma-Aldrich). Latex beads were pre-warmed for 1 h at 37 °C in complete medium (10% FBS in DMEM) before the phagocytosis assays. The pre-warmed beads were added to macrophages (number ratio approximately 10:1) for 4 h at 37 °C. The phagocytosis was terminated by the addition of 1 ml of pre-cooled PBS. Macrophages were harvested and analysed by flow cytometry.

### Gene expression analysis

TRIzol reagent (Invitrogen) was used to prepare total RNA from macrophages or tissue samples. Total RNA (1.5 μg) was reverse transcribed using a 5× All-In-One RT MasterMix (abm Cat#G486 Code Q111-02) kit. GAPDH was used as the normalisation gene. Q-PCR assays were carried out with a CFX96 real-time PCR detection system (Bio-Rad) using a Q-PCR kit (Vazyme Biotech). The comparative threshold method for relative quantification was used, and the results are expressed as -fold changes. The primers were synthesised by Invitrogen.

### Western blotting

Cells were collected and washed twice with PBS, and protein was extracted by whole-cell lysis with a kit purchased from Beyotime (Haimen, Jiangsu, China) that contained protease and phosphatase inhibitors. Centrifugation at 4 °C removed cellular debris, and the protein concentration was determined by a Pierce BCA assay. The protein content was electrophoresed on a 10% SDS-PAGE gel and then immunoblotted on a polyvinylidene fluoride membrane (American Biosciences). Antibodies against PARP (9532S, Cell Signaling Technology), PCNA (2586, Cell Signaling Technology), Caspase-9 (9508S, Cell Signaling Technology), and β-actin (KC-5A08, Kangchen Biotech) were used.

### Flow cytometry analysis

For apoptosis analysis, cells were stained with PE-Annexin V in the presence of 7-AAD using an Annexin V apoptosis detection kit according to the manufacturer’s instructions (BD Pharmingen, San Diego, CA).

For evaluation of macrophage phenotypes, Raw264.7 cells were incubated with a PE-conjugated anti-mouse CD206 (Clone M1) antibody, PE-conjugated anti-mouse MHCII antibody, PE-conjugated anti-mouse PDL1 antibody, PerCP-conjugated anti-mouse CD40 antibody, PE-Cy7-conjugated anti-mouse CD86 antibody, or PE-conjugated anti-mouse CD80 antibody for 30 min on ice followed by flow cytometry detection.

For cell cycle analysis, cells were collected and fixed with pre-cooled 70% ethanol at 4 °C for 2 h. The fixed cells were washed with PBS and stained with a PI working solution (50 μg/ml PI and 50 μg/ml RNase A) at room temperature for 30 min in the dark followed by flow cytometry detection.

### Construction of chimeric antigen receptors (plenti-CAR-HER2-CD147)

CAR-HER2-CD147 consists of an anti-hHER2 scFv that was derived from the A21 mouse hybridoma,^25,26^ the hinge region of mouse IghG1 (Gene ID: 16017, aa 98−110), and the transmembrane and intramembrane regions of the mouse CD147 molecule. The anti-hHER2 scFv encoding amino acid sequences was reverse translated, codon optimised, and synthesised as a single construct (Genscript, Jiangsu, China). The exact sequence of the CD147 molecule included in CAR-HER2-CD147 corresponds to the GenBank identifier NM_009768.2. The sequence includes all amino acids starting with the amino acid sequence MAALWP and continuing to the carboxy terminus of the protein. The signal peptide was derived from interferon gamma receptor 1 (IFNgR1, Gene ID: 15979). To facilitate the measurement of transfection efficiency, a myc-tag was inserted in front of the anti-hHER2 scFv. Homologous recombination was used to insert all fragments into the lentiviral vector pLenti6/V5-D-TOPO®.

### Tumour model and infusion of macrophages

All animals were assessed to be healthy and free of disease prior to tumour implantation. Mice were anaesthetised with an intraperitoneal injection of 70 mg/kg pelltobarbitalum natricum, and then 200,000 tumour cells in a volume of 20 μl were injected into the mammary fat pads of BALB/c mice or BALB/c nude mice. On days 8 and 15 after tumour cell inoculation, 1 × 10^6^ Raw264.7 cells were injected intravenously (i.v.). Tumour bioluminescence was analysed using the In Vivo Imaging System (IVIS Lumina XR, Caliper Life Sciences) for the first time on day 7. Animals were humanely euthanized on day 30, and tumour tissues were harvested and weighed. For animal studies of CAR-147 effects using BALB/c mice, three treatment groups (PBS, control Raw264.7 cells, CAR-147 Raw264.7 cells) were used, each with five animals. The experiment was repeated, and data were pooled, including ten mice per group. For animal studies using BALB/c nude mice, three treatment groups (PBS, control Raw264.7 cells, CAR-147 Raw264.7 cells) were used, each with eight animals. Each experimental group was confined to a separate cage. Cages were selected for a particular treatment at random at the start of treatment (all treatments were started at the same time). All groups were assessed at the same time. Tumours were allowed to establish until they were palpable or detectable by bioluminescence prior to treatments. Animals were euthanized by carbon dioxide asphyxiation followed by cervical dislocation to ensure death.

### Preparation of tumour single-cell suspensions

Tumours from treated mice were cut into small pieces using scissors, followed by digestion with pre-warmed 0.1% collagenase I containing 75 µg/ml DNase I (1 h, 37 °C). The digested tissue samples were filtered using a 40-μm cell strainer. Then, a red blood cell lysis buffer was used to remove red blood cells at RT for 2 min. The single-tumour cell suspensions were analysed by flow cytometry.

### Cytokine assay by ELISA

IL-1β, IL-6, IL-10, IL-12, TNFα and IFNγ concentrations in cell culture supernatants, blood samples and tumour homogenates were assessed using ELISA kits according to the manufacturer’s protocols.

### In vivo near-infrared fluorescence imaging

To investigate the localisation of infused macrophages in tumour-bearing mice, 10^6^ control Raw264.7 cells or 10^6^ CAR-147 Raw264.7 cells stained with the near-infrared fluorescent probe DiR (Yeasen Biotech, China) were injected intravenously. Ten mice were used (*n* = 5 control Raw264.7 group versus *n* = 5 CAR-147 Raw264.7 group). At different time intervals, the mice were anaesthetised and imaged using the In Vivo Imaging System (IVIS Lumina XR, Caliper Life Sciences).

### Three-dimensional multicellular sphere culture (MCS)

In total, 4000 MDA-MB-453 tumour cells and 1000 PMA-treated THP-1 macrophages were added to the 96-well Clear Round-bottom Ultra Low-attachment Microplate (Corning, USA) for 72 h at 37 °C. The MCSs were monitored with a microscope, and uniform tumour spheroids were selected for subsequent studies. To study MCS penetration by T lymphocytes, Jurkat T cells were stained with 5 μM CFDA-SE (carboxyfluorescein diacetate succinimidyl ester) (Beytotime, Jiangsu, China) for 15 min at 37 °C in PBS. Labelling was stopped by adding cold complete medium and washing three times. Then, 50,000 Jurkat T cells were incubated with a spheroid for 20 h. After washing and fixing in 4% paraformaldehyde, the tumour spheroids were imaged under a Nikon confocal microscope (Nikon TE2000U, Tokyo, Japan).

### Statistical analysis

Data are expressed as the mean ± SEM. Statistical analysis was performed by Student’s *t* test when only two value sets were compared. One-way ANOVA followed by Dunnett’s test was used when the data involved three or more groups. *P* < 0.05, *P* < 0.01 or *P* < 0.001 were considered statistically significant and indicated by *, ** or ***, respectively.

## Results

### Construction of CAR-147 targeting HER2

**Fig. 1:**
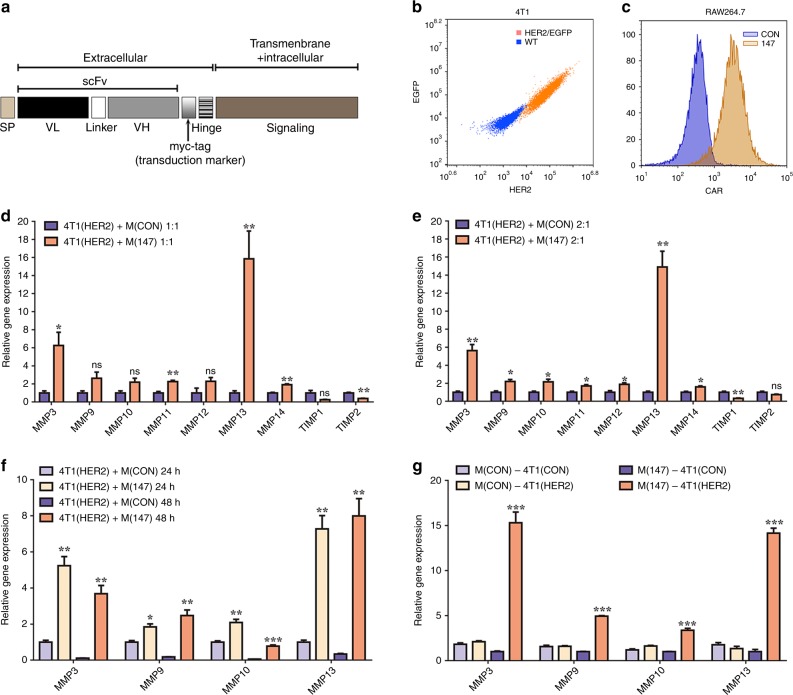
Construction of the chimeric antigen receptor CAR-147 targeting HER2. **a** The design of a chimeric antigen receptor (CAR-147) for macrophages is shown. **b** Human HER2 surface expression on 4T1 cells was measured. **c** CAR-147 surface expression on Raw264.7 cells was measured. **d** Control Raw264.7 (CON) cells and CAR-147 Raw264.7 (147) cells were cocultured with HER2-4T1 (HER2) cells at a ratio of 1:1 for 24 h, and the expression of MMP genes was assessed by RT-qPCR (*n* = 6). **e** Control Raw264.7 (CON) cells and CAR-147 Raw264.7 (147) cells were cocultured with HER2-4T1 (HER2) cells at a ratio of 1:2 for 24 h, and the expression of MMP genes was assessed by RT-qPCR (*n* = 6). **f** Control Raw264.7 (CON) cells and CAR-147 Raw264.7 (147) cells were cocultured with HER2-4T1 (HER2) cells at a ratio of 1:2 for 24 or 48 h, and the expression of MMP genes was assessed by RT-qPCR (*n* = 6). **g** Control Raw264.7 (CON) cells or CAR-147 Raw264.7 (147) cells were cocultured with wild-type 4T1 (CON) cells or HER2-4T1 (HER2) cells at a ratio of 1:1 for 24 h, and the expression of MMP genes was assessed by RT-qPCR (*n* = 6). All values are expressed as the mean ± SEM. *P < 0.05, **P < 0.01, and ***P < 0.001

To breakdown the “physical barrier” of the tumour matrix, we selected CD147, a membrane molecule that is essential for ECM remodelling via the expression of MMPs.^27^ According to the chimeric antigen receptor design principle, we generated a modified CAR-147 construct for macrophages (the detailed structure is shown in Fig. 1a). Briefly, CAR-147 is composed of a single-chain antibody fragment targeting human HER2, the hinge region of mouse IghG1, and the transmembrane and intracellular regions of the mouse CD147 molecule. The HER2-scFv sequence was designed by Sangon Biotech based on reports in the NCBI database (PDB: 3H3B_D). To verify that the constructed chimeric antigen receptor system can activate internal signalling after stimulation by HER2 and affect the expression of the downstream signal, we established stable clones of 4T1 cells overexpressing human HER2-EGFP (HER2-4T1) (Fig. 1b) and Raw264.7 macrophages expressing CAR-147 (Fig. 1c). HER2-EGFP includes the replacement of the HER2 intracellular region with EGFP to avoid the effect of HER2 on the 4T1 cells. Control macrophages and CAR-147 macrophages were directly cocultured with HER2-4T1 cells. After coculture for 24 h, the macrophages from each group were collected to analyse the expression of MMPs and TIMPs by real-time PCR. As shown in Fig. 1d, the expression of multiple MMPs (MMP3, MMP11, MMP13, and MMP14) was significantly upregulated in the CAR-147 macrophages at an E:T ratio of 1:1. When the E:T ratio was 2:1, the expression of MMP9, MMP10 and MMP12 was also upregulated (Fig. 1e), indicating that the increase in MMP expression in the CAR-147 macrophages was related to tumour antigen stimulation. Increased MMP expression was also detected after coculture for 48 h (Fig. 1f). In parallel, the expression levels of MMPs in CAR-147 macrophages cocultured with wild-type (WT) 4T1 cells were not different from those in control macrophages, indicating that the chimeric antigen receptor could specifically recognise the antigen (Fig. 1g). These data demonstrated that CAR-147 could specifically recognise the antigen HER2 and effectively activate the expression of MMPs in macrophages.

To assess the effect of CAR-147 on macrophage phenotype, flow cytometry was used to analyse the expression of membrane molecules (MHCII, CD206, PDL1, CD40, CD80, and CD86) in macrophages after coculture. We found that CAR-147 had no effect on macrophage membrane molecule expression, except for inducing increased expression of CD80 (Supplementary Fig. 1A). In addition, a phagocytosis assay using fluorescent red latex beads and a reactive oxygen species assay using the DCFH-DA probe showed that CAR-147 did not affect phagocytosis (Supplementary Fig. 1B) or ROS production in macrophages (Supplementary Fig. 1C), respectively. Furthermore, we evaluated the release of inflammatory cytokines from control macrophages and CAR-147 macrophages after coculture for 48 h. The results showed that there were no differences in the secretion of IL-1β, IL-6, IL-10, IL-12, TNFα or IFNγ (Supplementary Fig. 1D). These data indicate that CAR-147 specifically activates MMP expression without affecting other functions, such as phagocytosis, ROS production, and inflammatory cytokine secretion.

### CAR-147 macrophages inhibit tumour growth in vivo

We next investigated the effects of CAR-147 macrophages on tumour cell growth in vitro. An apoptosis assay showed that there was no difference in apoptosis in HER2-4T1 cells after coculture with control macrophages or CAR-147 macrophages (Supplementary Fig. 2A), which was consistent with the results for western blotting detection of apoptosis-related proteins (PARP and Caspase-9) (Supplementary Fig. 2B). Cell cycle analysis by PI staining revealed that compared with control macrophages, CAR-147 macrophages did not affect the cell cycle in HER2-4T1 cells (Supplementary Fig. 2C). The protein expression level of PCNA, which indicates the cell proliferation ability of HER2-4T1 cells, was not changed (Supplementary Fig. 2B). The effect of CAR-147 macrophages on HER2-4T1 cell invasion was examined with Matrigel, a reconstituted ECM. As shown in Supplementary Fig. 2D, compared with control macrophages, CAR-147 macrophages had no effect on the invasion ability of HER2-4T1 cells. Therefore, CAR-147 macrophages did not inhibit the growth of tumour cells in vitro.

**Fig. 2:**
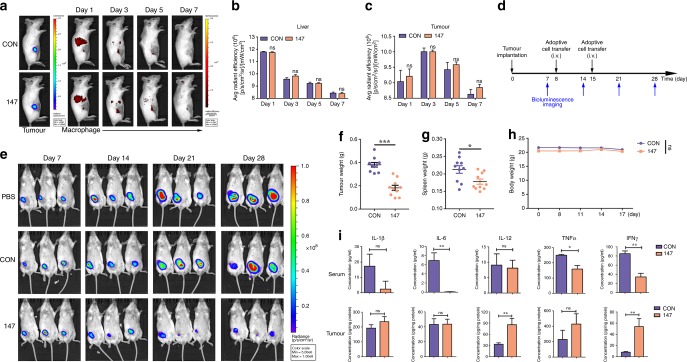
The effect of CAR-147 macrophages on tumour growth in vivo. **a** In vivo imaging of luciferase-expressing HER2-4T1 tumour-bearing mice after intravenous injection of DiR-labelled Raw264.7 cells. **b** Quantitative analysis of the DiR fluorescence signal in the liver (*n* = 5). **c** Quantitative analysis of the DiR fluorescence signal at the tumour site (*n* = 5). **d** The experimental schedule for tumour implantation, macrophage infusion and bioluminescence imaging (BLI) monitoring. **e** BLI was performed on day 7 to assess tumour burden. Control Raw264.7 (CON) cells (1 × 10^6^), CAR-147 Raw264.7 (147) cells (1 × 10^6^), or PBS were injected i.v. on day 8 and day 15, and mice were followed with serial BLI. **f** The weight of tumours from control Raw264.7 cell- or CAR-147 Raw264.7 cell-treated mice was analysed. The data presented are pooled from two independent experiments. Each symbol indicates one mouse (*n* = 10). **g** The spleen weight of tumour-bearing mice was analysed. **h** The body weight of tumour-bearing mice was measured every 3 days. **i** Blood serum samples and tumour homogenates were collected from control Raw264.7 cell- or CAR-147 Raw264.7 cell-treated mice and analysed for proinflammatory cytokine release (IL-6, IFNγ, TNFα, IL-1β, IL-12) by ELISA (*n* = 5). All values are expressed as the mean ± SEM. *P < 0.05, **P < 0.01, and ***P < 0.001

To further examine the effect of the designed chimeric antigen receptor on solid tumours in vivo, we established a mouse model of breast cancer with orthotopically transplanted HER2-4T1 cells. To analyse the tissue-distribution and time-course changes of infused macrophages, 10^6^ EGFP^+^ macrophages or 10^6^ DiR-labelled macrophages were injected intravenously for flow analysis or in vivo imaging, respectively. In normal mice, in vivo imaging showed that the DiR-labelled macrophages mainly accumulated in the liver and were almost completely gone by 144 h post-infusion (Supplementary Fig. 3A, B). In tumour-bearing mice, the DiR-labelled macrophages were detected at the tumour site on day 1 post-infusion, and the maximum signal was detected on day 3, as visualised by fluorescence imaging (Fig. 2a–c). The infiltration of the infused macrophages into tumour tissue was also detected by flow cytometry, and the corresponding gating strategy is presented in Supplementary Fig. 3C. The results showed that CAR-147 had no effect on the infiltration of infused macrophages into tumours (Fig. 2c and Supplementary Fig. 3D). Furthermore, we analysed the phenotypes of infused macrophages in tumours. No differences were observed between control macrophages and CAR-147 macrophages (Supplementary Fig. 3E).

The tumour microenvironment may contribute to the progressive differentiation of infused macrophages. However, compared with no treatment, macrophage infusion did not promote tumour growth (Fig. 2e). Control macrophages and CAR-147 macrophages were injected intravenously on the 8th and 15th day post tumour implantation (Fig. 2d). Beginning on the 7th day, tumour burden was measured by the In Vivo Imaging System, and we observed that the CAR-147 macrophages effectively inhibited tumour growth compared with the control macrophages (Fig. 2e, f) and reduced spleen weight (Fig. 2g), while body weight (Fig. 2h) was unaffected in both groups. To further analyse whether CAR-147 macrophage reinfusion causes cytokine storms like CAR-T cell infusion does, we detected the cytokines IL-1β, IL-6, IL-12, TNFα, and IFNγ in serum samples and tumour homogenates. Interestingly, the results showed that the IFNγ, TNFα, and IL-6 levels in the serum of the CAR-147 macrophage group were decreased compared with that of the control group (Fig. 2i), while the IL-12 and IFNγ levels were increased in tumour tissue samples from the CAR-147 macrophage-treated mice (Fig. 2i). Overall, CAR-147 macrophages significantly inhibited tumour growth in the 4T1 breast cancer mouse model but might not cause cytokine release syndrome.

### CAR-147 macrophages can promote T-cell infiltration into tumours

**Fig. 3:**
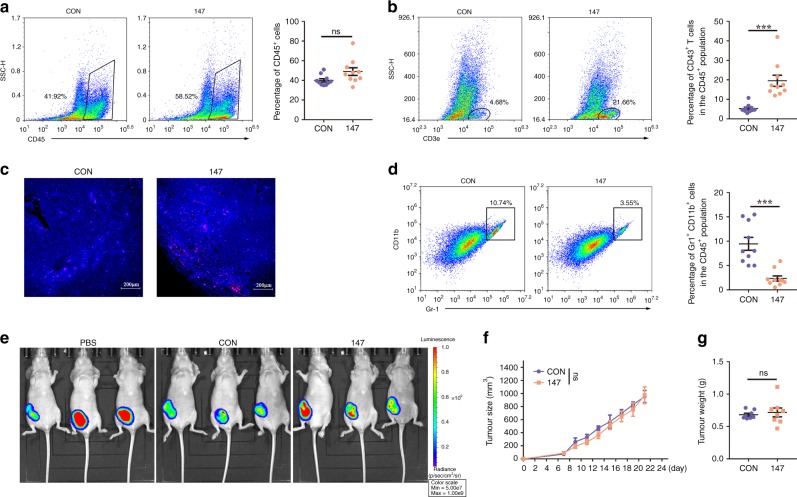
CAR-147 macrophages can promote T-cell infiltration into tumours. **a** Flow cytometric analysis and quantification of CD45^+^ tumour-infiltrating leucocytes (TILs) (*n* = 10). **b** Flow cytometric analysis and quantification of CD3^+^ T cells in the TIL population (*n* = 10). **c** Immunofluorescence analysis of CD3^+^ T-cell infiltration in tumour tissues from control Raw264.7 cell- and CAR-147 Raw264.7 cell-treated animals. **d** Flow cytometric analysis and quantification of MDSCs (Gr-1^+^CD11b^+^) in the TIL population (*n* = 10). **e** In total, 2 × 10^5^ HER2-4T1 cells were transplanted into BALB/c nude mice. BLI was performed to assess tumour burden. **f** Tumour growth from control Raw264.7 cell- or CAR-147 Raw264.7 cell-treated BALB/c nude mice was analysed by measuring volumes every 2 days. **g** The weight of tumours from control Raw264.7 cell- or CAR-147 Raw264.7 cell-treated BALB/c nude mice was analysed. Each symbol indicates one mouse (*n* = 8). All values are expressed as the mean ± SEM. *P < 0.05, **P < 0.01, and ***P < 0.001

The purpose of the CAR-147 macrophages we designed is to remodel the tumour ECM, destroying the physical barrier in solid tumours to promote T-cell infiltration and thus inhibit tumour growth. We have shown remarkable antitumoural effects of CAR-147 macrophages on 4T1 tumour-bearing mice. For further study, we performed multicolour flow cytometric analysis to investigate immune cell infiltration and phenotypes. As shown in Fig. 3a, no difference was observed in the number of CD45^+^ tumour-infiltrating leucocytes (TILs). It is noteworthy that CAR-147 macrophage-treated tumours exhibited significantly more CD3^+^ T-cell content (Fig. 3b, c) and less MDSC content in the TIL population (Fig. 3d) than control macrophage-treated tumours. However, there were no significant differences in DC (MHCII^+^CD11c^+^) or NK (NK1.1^+^) cell infiltration (Supplementary Fig. 4A, B). The number and phenotype of tumour-associated macrophages (TAMs) were unaffected in both groups (Supplementary Fig. 4C, D). To characterise T-cell function in tumours, we first examined the percentage of CD8^+^ T cells in the CD3^+^ T-cell population and found that this percentage was not affected (Supplementary Fig. 4E). The expression levels of CD44 and CD62L can be used to identify the activation status of T cells. The percentages of CD44^high^CD62L^low^ effector T cells in the CD3^+^ T-cell population remained unchanged (Supplementary Fig. 4F). Furthermore, the expression of the degranulation marker CD107a and T-cell exhaustion marker PD-1 was analysed. No differences were observed between the two groups (Supplementary Fig. 4G, H). We analysed the correlation between the percentage of CD3^+^ T cells and tumour weight in the two groups. The two factors showed a stronger correlation in the CAR-147 macrophage group (Supplementary Fig. 5A), suggesting that T cells play an important role in the anti-tumour effect of CAR-147 macrophages. We next addressed the extent to which the anti-tumour effect of CAR-147 macrophages depended on host T cells. Thus, we inoculated HER2-4T1 cells into syngeneic BALB/c nude mice. The course of macrophage infusion treatment that was efficacious in WT BALB/c mice had no effect on tumour growth in the T-cell-deficient nude mice (Fig. 3e–g). These results suggest that CAR-147 macrophages promote enhanced CD3^+^ T-cell mobilisation in breast cancer, which is clearly required for the therapeutic effect of CAR-147 macrophage infusion.

### CAR-147 macrophages reduce extracellular matrix deposition

**Fig. 4:**
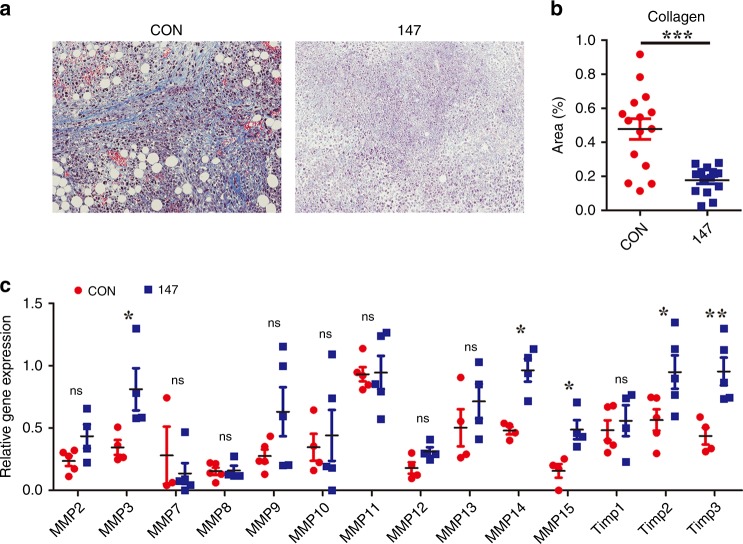
CAR-147 macrophages reduce extracellular matrix deposition. **a** Representative images of tumour sections stained with Masson’s trichrome to detect extracellular matrix deposition (blue). Scale bar, 100 μm. **b** Quantification of the extracellular matrix (*n* = 15). **c** Analysis of MMP gene expression in tumour tissues from control Raw264.7 cell- and CAR-147 Raw264.7 cell-treated animals by RT-qPCR (*n* = 5). All values are expressed as the mean ± SEM. *P < 0.05, **P < 0.01, and ***P < 0.001

To further assess whether CAR-147 macrophages act on the ECM and disrupt this “physical barrier”, we used Masson’s trichrome staining to analyse the collagen content in tumour tissue. Consistent with our premise, collagen content was significantly reduced after CAR-147 macrophage treatment compared with control macrophage treatment (Fig. 4a, b). Next, we examined the expression of various MMPs and TIMPs in tumours. The results showed that there were no differences in the expression levels of most MMPs, except MMP3, MMP14 and MMP15 levels were upregulated in CAR-147 macrophage-treated mice (Fig. 4c). Extracellular matrix breakdown by MMPs is essential for tumour cell invasion and metastasis. Therefore, degradation of the matrix by CAR-147 macrophages is likely to promote tumour cell metastasis. Then, we assessed the effect of CAR-147 macrophages on tumour metastasis. The results showed that CAR-147 macrophages did not promote tumour metastasis while inhibiting tumour growth (Supplementary Fig. 5B). In summary, the infusion of CAR-147 macrophages can degrade the dense collagen-based matrix that surrounds tumours, which may require the involvement of MMP3, MMP14 and MMP15.

### CAR-147 macrophages facilitate T-cell infiltration in three-dimensional multicellular sphere models of human breast cancer

**Fig. 5:**
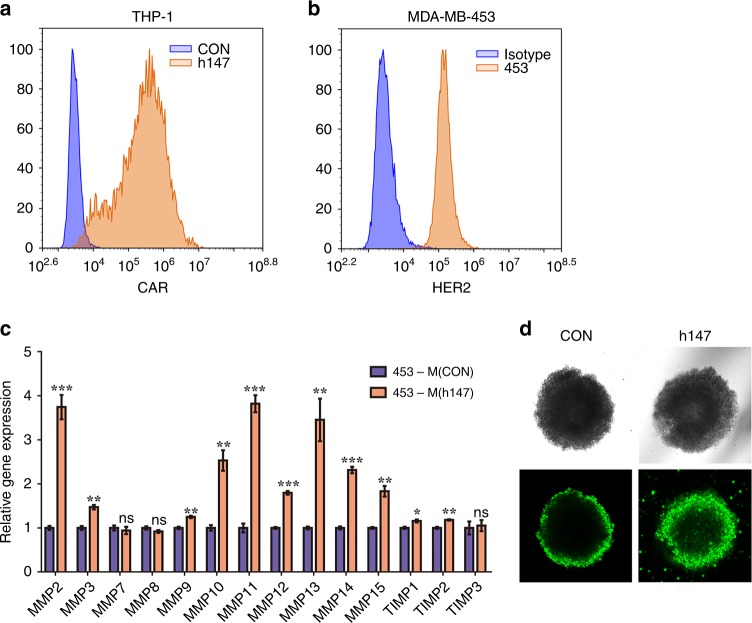
CAR-147 macrophages facilitate T-cell infiltration into 3D human tumour spheroids. **a** CAR-h147 surface expression on THP-1 cells. **b** Detection of HER2 expression on MDA-MB-453 cells by flow cytometry. **c** PMA-treated CAR-h147 THP-1 cells were cocultured with MDA-MB-453 tumour cells at a ratio of 1:1 for 48 h, and the expression of MMP genes was assessed by RT-qPCR (*n* = 6). **d** Confocal microscopy images of MDA-MB-453/THP-1 spheroids treated with CFSE-labelled Jurkat T cells for 20 h. Scale bars, 100 μm. All values are expressed as the mean ± SEM. *P < 0.05, **P < 0.01, and ***P < 0.001

To determine whether this treatment strategy works with human macrophages, we constructed a chimeric antigen receptor containing the internal signalling domain of the human CD147 molecule (CAR-h147). The human monocytic leukaemia cell line THP-1 shares many properties with human monocyte-derived macrophages after stimulation with phorbol-12-myristate 13-acetate (PMA) for 24 h.^28^ Here, we examined the expression of MMPs in PMA-treated THP-1 macrophages expressing CAR-h147 (Fig. 5a) after coculture with HER2^+^ human breast cancer MDA-MB-453 tumour cells (Fig. 5b). The results showed that the expression of multiple MMPs (MMP2, MMP3, MMP9, MMP10, MMP11, MMP12, MMP13, MMP14, and MMP15) in the CAR-h147 THP-1 macrophages was upregulated after coculture compared with that in control THP-1 macrophages (Fig. 5c). Because 2D cultures cannot be used for cell infiltration studies, we performed three-dimensional multicellular sphere culture (MCS). It was observed that Jurkat T cells were able to penetrate deeper into the MCS formed by MDA-MB-453 tumour cells and CAR-h147 THP-1 macrophages compared to that formed by tumour cells and control THP-1 macrophages (Fig. 5d). These ex vivo studies initially demonstrated that CAR-147 macrophages facilitated T-cell infiltration in a human tumour model.

## Discussion

Tumours and stromal cells produce and assemble collagen, proteoglycans, and other molecules to form rigid ECMs and establish physical barriers that reduce the spread of therapeutic agents to tumour cells. The potential problem with many novel anticancer strategies is the inability to penetrate the tumour stroma, which is one of the reasons why CAR-T cell therapy is not effective in solid tumours.

In stroma-rich pancreatic ductal adenocarcinoma, blood vessel density is significantly reduced, and blood vessels are embedded within the matrix, resulting in the inability of therapeutic agents to reach the tumour site.^29^ Hélène Salmon et al. reported that the density and orientation of the stromal ECM affected the localisation and migration of T cells in human non-small cell lung cancer tumours, in which more T cells were observed in regions of loose fibronectin and collagen and these cells migrated in a linear manner along fibronectin fibres.^8^ Lysyl oxidase (LOX) and LOX-like (LOXL) catalyse the cross-linking and stabilisation of collagen and have high activity in tumours. In a mouse model of pancreatic cancer, LOX inhibition was shown to suppress metastasis and promote the efficacy of gemcitabine.^30^ In addition, the ECM provides essential signals to promote tumour cell growth and inhibit apoptosis. Some studies have reported that the signals initiated by collagen IV are important cues for the survival and growth of tumour cells in the liver. Accordingly, finding an effective treatment strategy for targeting the ECM is necessary.

In the tumour microenvironment, macrophages can account for 50% of the leucocytes present, most of which are recruited from the peripheral blood.^31^ It has been observed that macrophage infusion can improve liver fibrosis in animal models.^19^ These data suggest that using modified macrophages to target the tumour ECM may be a novel and effective strategy. In the 1980s, clinical trials in cancer patients studying the adoptive transfer of macrophages generated from blood monocytes were carried out^32^ and demonstrated the safety and feasibility of macrophage reinfusion in patients. In our study, infused macrophages accumulated mainly in the liver and tumour site and disappeared gradually over 7 days after infusion. These data suggest that macrophages can induce durable anti-tumour effects in a very short time period, indicating that adoptive macrophage therapy is a controlled and safe therapeutic strategy.

In our study, we modified macrophages to target the tumour ECM based on the chimeric antigen receptor structure used in CAR-T cell therapy. The chimeric antigen receptor designed for macrophages mainly contained two regions: one was an extracellular region, a single-chain variable region moiety (scFv) that recognised HER2, which is an important biomarker of breast cancer, and the other was the intracellular region of CD147, which is used to activate the expression of MMPs in macrophages. CD147, also known as ECM metalloproteinase inducer (EMMPRIN), is a member of the class of transmembrane glycoproteins that plays important roles in regulating the synthesis and expression of cellular MMPs. It has been reported that high expression of CD147 will greatly increase the quantity and activity of MMPs, thereby increasing the degradation rate of the basement membrane and destroying this natural mechanical barrier. Our data showed that CAR-147 can upregulate the expression of multiple MMPs in macrophages after coculture with HER2-4T1 cells in vitro. However, except for CD80, membrane surface molecules did not exhibit changed expression, and phagocytosis, ROS production, and inflammatory cytokine secretion in macrophages were also unaffected. Additionally, CAR-147 macrophages did not affect tumour cell growth in vitro compared with control macrophages.

In the HER2-4T1 tumour model, we observed that the infusion of CAR-147 macrophages significantly inhibited tumour growth. Since it was demonstrated that CAR-147 macrophages did not affect the growth of tumour cells in vitro, we tested whether the effect of inhibiting tumours was dependent on autologous T cells as we predicted. The results showed that the proportion of T cells in the CAR-147 macrophage-treated tumours was approximately four times higher than that in the control macrophage-treated tumours, while the same macrophage infusion treatment process had no effect on tumour growth in T-cell-deficient nude mice. These data indicated that CAR-147 macrophages promote T-cell infiltration, which is clearly required for the therapeutic effect. In the current study on tumour immunotherapy, it is still a big challenge to reconstruct the immune system of immunodeficient mice with human immune cells. Therefore, it is difficult to test the utility of CAR-147 human macrophages in immune-compromised mice with human HER2^+^ breast cancer tumours. However, we performed a 3D-culture model to preliminarily demonstrate that this treatment strategy can also be effective in human breast cancer tumours. Some studies have reported that adequate T-cell infiltration in tumours is a prerequisite for sensitivity to immune checkpoint blockade (ICB) therapy.^33^ This observation may suggest that CAR-147 macrophages can overcome tumour resistance to ICB therapy by increasing T-cell infiltration. After macrophage infusion, the collagen content in tumours was significantly reduced, while the expression of MMP3, MMP14 and MMP15 was increased. CAR-147 macrophages may require multiple MMPs to exert an anti-tumour function. When designing chimeric antigen receptors for different types of tumours, the effects of different MMPs should be considered.

Cytokine release syndrome (CRS) is the most frequent toxic event in clinical trials when using CAR-T cells to treat haematological malignancies, and severe CRS can be fatal. IL-6 is the main inflammatory mediator of CRS. It has long been thought that CAR-T cells cause CRS, but some studies have reported that this side effect is largely attributed to macrophages.^34,35^ Interestingly, in our study, we found that the levels of inflammatory cytokines (IL-6, TNFα, IFNγ) in CAR-147 macrophage-treated mice were lower than those in control mice. However, cytokines are important mediators of the immune system involved in the exertion of anti-tumour effects. We further analysed changes in inflammatory cytokines in tumours after macrophage infusion. CAR-147 macrophages significantly increased the levels of IL-12 and IFNγ, which can exert potent anti-tumour responses, in the tumour tissue. These findings suggest that the effects of CAR-147 macrophages are local and relatively tumour specific.

In conclusion, we provide a novel approach to target the tumour ECM by engineered macrophage infusion. Our data showed that after recognition of the antigen HER2, CAR-147 macrophages can increase the expression of MMPs to degrade the tumour ECM, which can promote T-cell infiltration and inhibit tumour growth. CAR-147 macrophage treatment combined with other anti-tumour therapies, such as CAR-T cells, ICB, and chemotherapeutic drugs, may be relatively effective in the elimination of cancer cells with minimal side effects.

## Supplementary information

Supplementary Information

## Data Availability

All data are available via the corresponing author.
