# Supplementary material for: Chimeric antigen receptor macrophage therapy for breast tumours mediated by targeting the tumour extracellular matrix
Source: Br J Cancer. 2019 Oct 1;121(10):837–45. doi: 10.1038/s41416-019-0578-3 (PMC6889154; doi:10.1038/s41416-019-0578-3)
Supplement: Supplementary file 1 — Supplementary Information [file 41416_2019_578_MOESM1_ESM.docx]

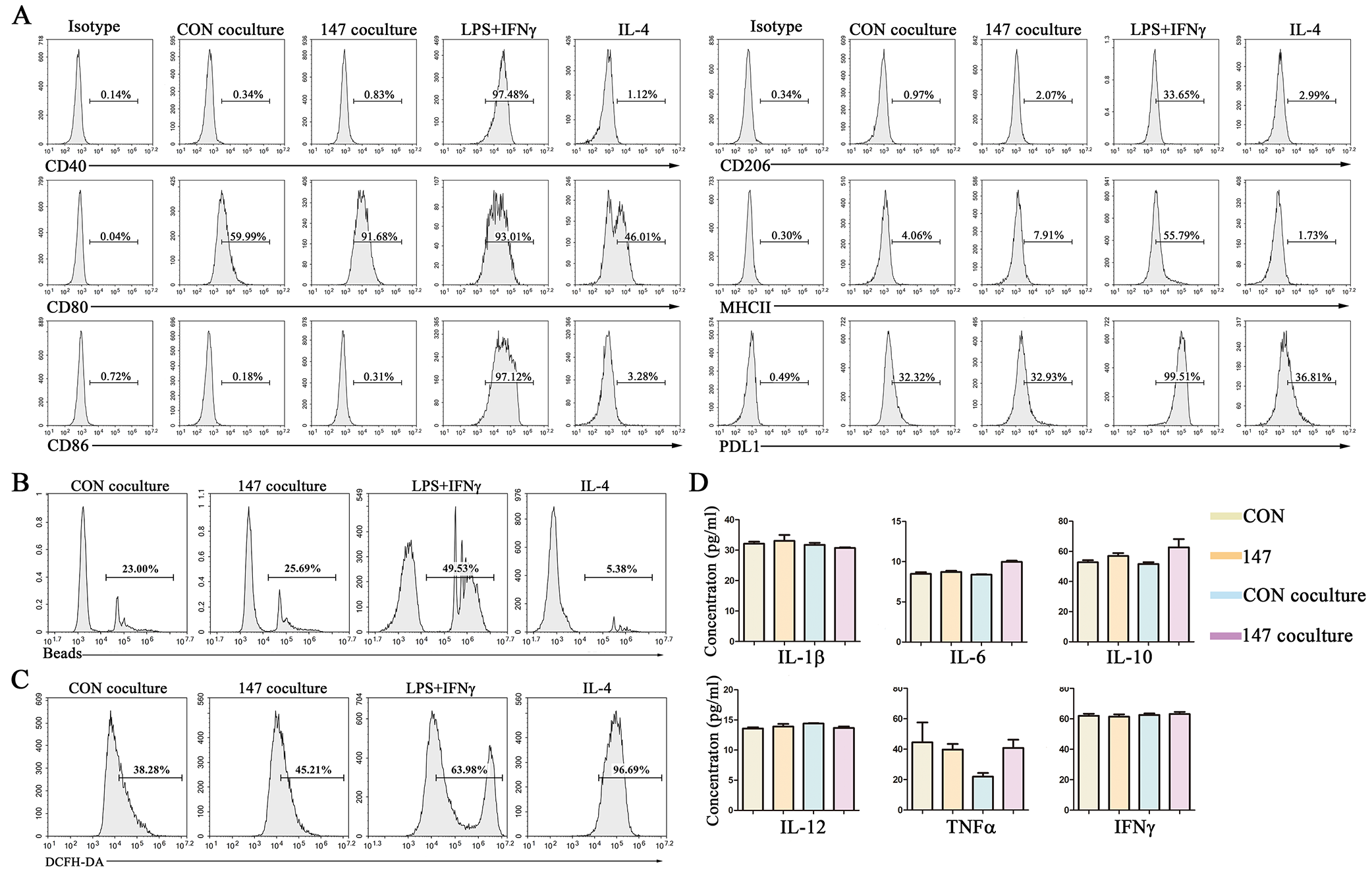


**Supplementary Figure 1. The effect of CAR-147 on the phenotype and function of macrophages.** **A.** Flow cytometry analysis of the phenotype (CD40, CD80, CD86, CD206, MHCII, and PDL1) of Raw264.7 cells cocultured with HER2-4T1 cells for 48 h or stimulated with LPS (100 ng/ml) and IFNγ (20 ng/ml) or IL-4 (10 ng/ml) for 48 h. **B.** Phagocytic abilities of Raw264.7 cells cocultured with HER2-4T1 cells for 48 h or stimulated with LPS+IFNγ for 48 h or IL-4 for 48 h, as determined by analysis with latex beads. **C.** The level of ROS produced by Raw264.7 cells cocultured with HER2-4T1 cells for 48 h or stimulated with LPS+IFNγ or IL-4 for 48 h, as analysed by flow cytometry using a DCFH-DA probe. **D.** The levels of inflammatory cytokines (IL-6, IFNγ, TNFα, IL-1β, IL-10, and IL-12) in the cell culture supernatant of Raw264.7 cells alone or in the coculture system, as measured by ELISA. Data similar to the data shown in the figure were obtained in at least three independent experiments.


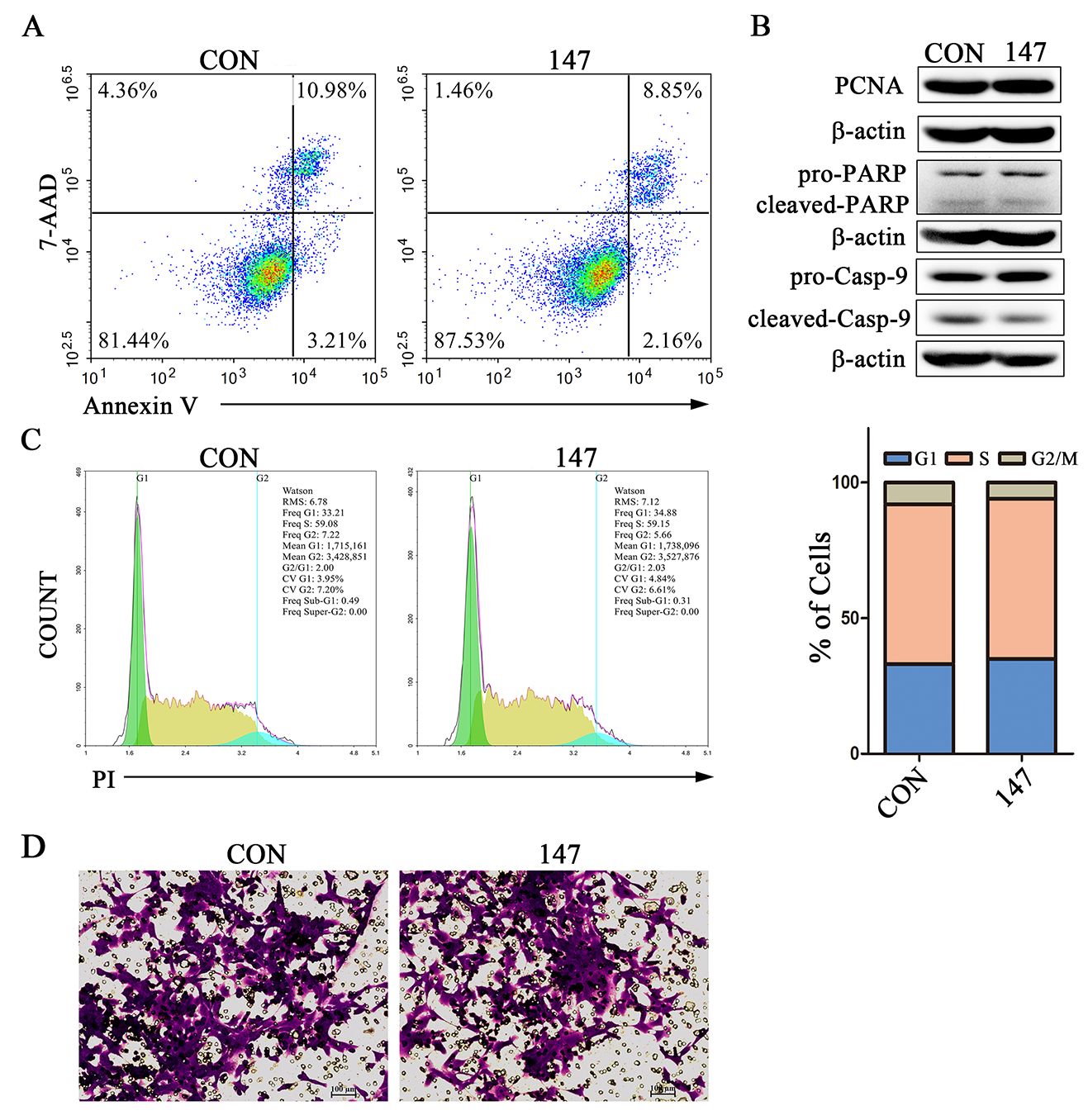


**Supplementary Figure 2. The effect of CAR-147 macrophages on tumour cells *in vitro.* A.** Flow cytometry analysis of apoptosis in HER2-4T1 cells after coculture with control Raw264.7 (CON) cells or CAR-147 Raw264.7 (147) cells via Annexin V/7-AAD staining. **B.** HER2-4T1 cells harvested after coculture with control Raw264.7 (CON) cells or CAR-147 Raw264.7 (147) cells and assessed for PCNA, PARP and Caspase-9 expression by western blotting. **C.** Cell cycle analysis of HER2-4T1 cells after coculture with control Raw264.7 (CON) cells or CAR-147 Raw264.7 (147) cells by flow cytometry analysis of propidium iodide (PI) staining. **D.** An invasion assay with HER2-4T1 cells after coculture with control Raw264.7 (CON) cells or CAR-147 Raw264.7 (147) cells evaluated by crystal violet staining. Scale bars, 100 μm. All values are expressed as the mean ± SEM. *P < 0.05, **P < 0.01; and ***P < 0.001. Data in the figure are presented as the representative result of at least three independent experiments.


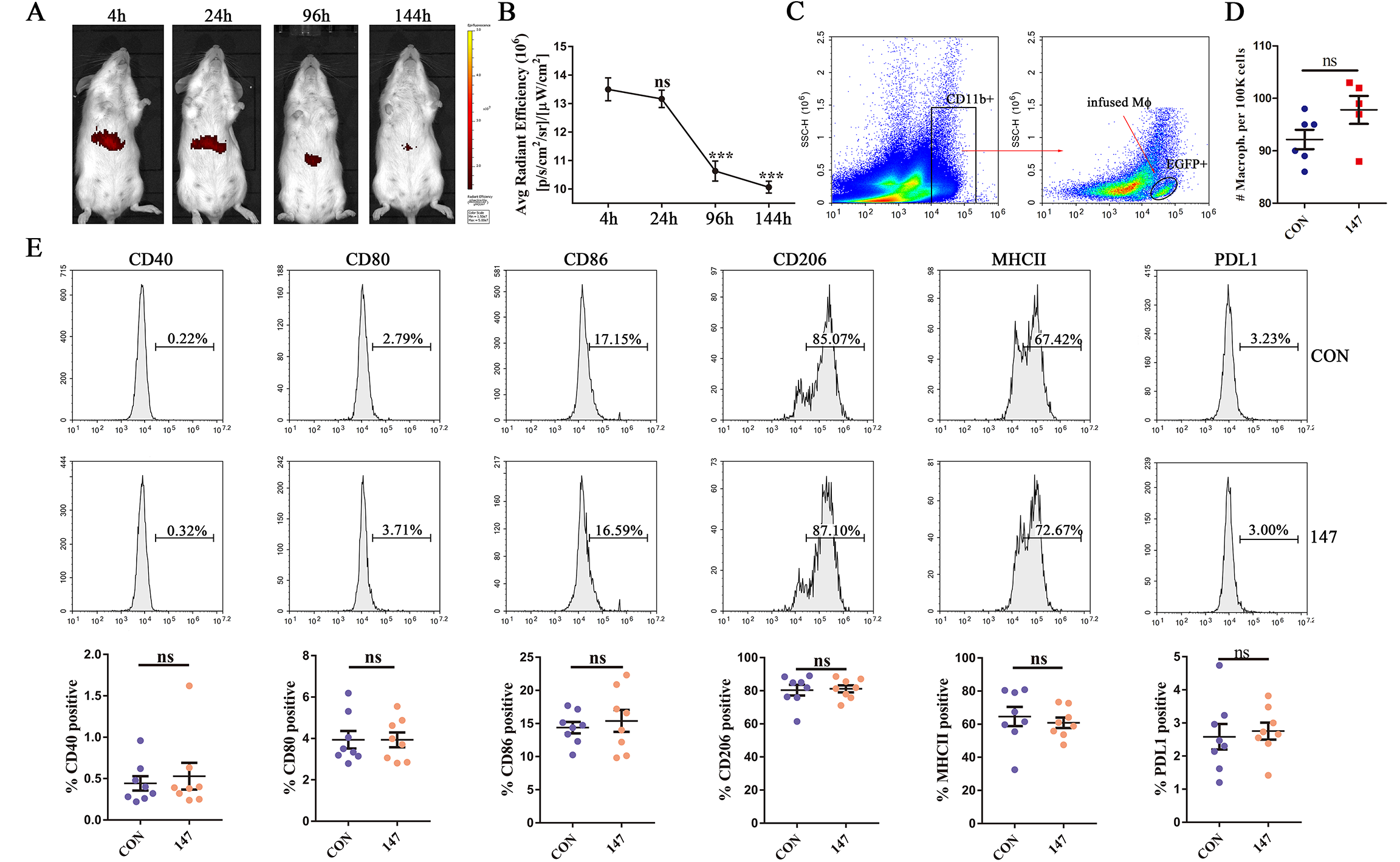


**Supplementary Figure 3. The localization and phenotypic analysis of infused macrophages *in vivo*. A.** Near-infrared imaging of normal mice at the indicated time post intravenous injection of DiR-labelled Raw264.7 cells. **B.** Quantitative analysis of the DiR fluorescence signal. The results are expressed as the mean fluorescence intensity ± SEM.; n = 5. Student’s t test. **p < 0.01, ***p < 0.001. **C.** Gating strategy to identify infused EGFP^+^ Raw264.7 cells. **D.** Control Raw264.7 (CON) and CAR-147 Raw264.7 (147) cell numbers per 100k cells in tumours as measured by flow cytometry 3 days after intravenous injection (n=6). **E.** Flow cytometry analysis of the phenotype (CD40, CD80, CD86, CD206, MHCII, and PDL1) of infused control Raw264.7 (CON) cells and CAR-147 Raw264.7 (147) cells isolated from HER2-4T1 tumours (n=8). All values are expressed as the mean ± SEM. *P < 0.05, **P < 0.01; and ***P < 0.001.


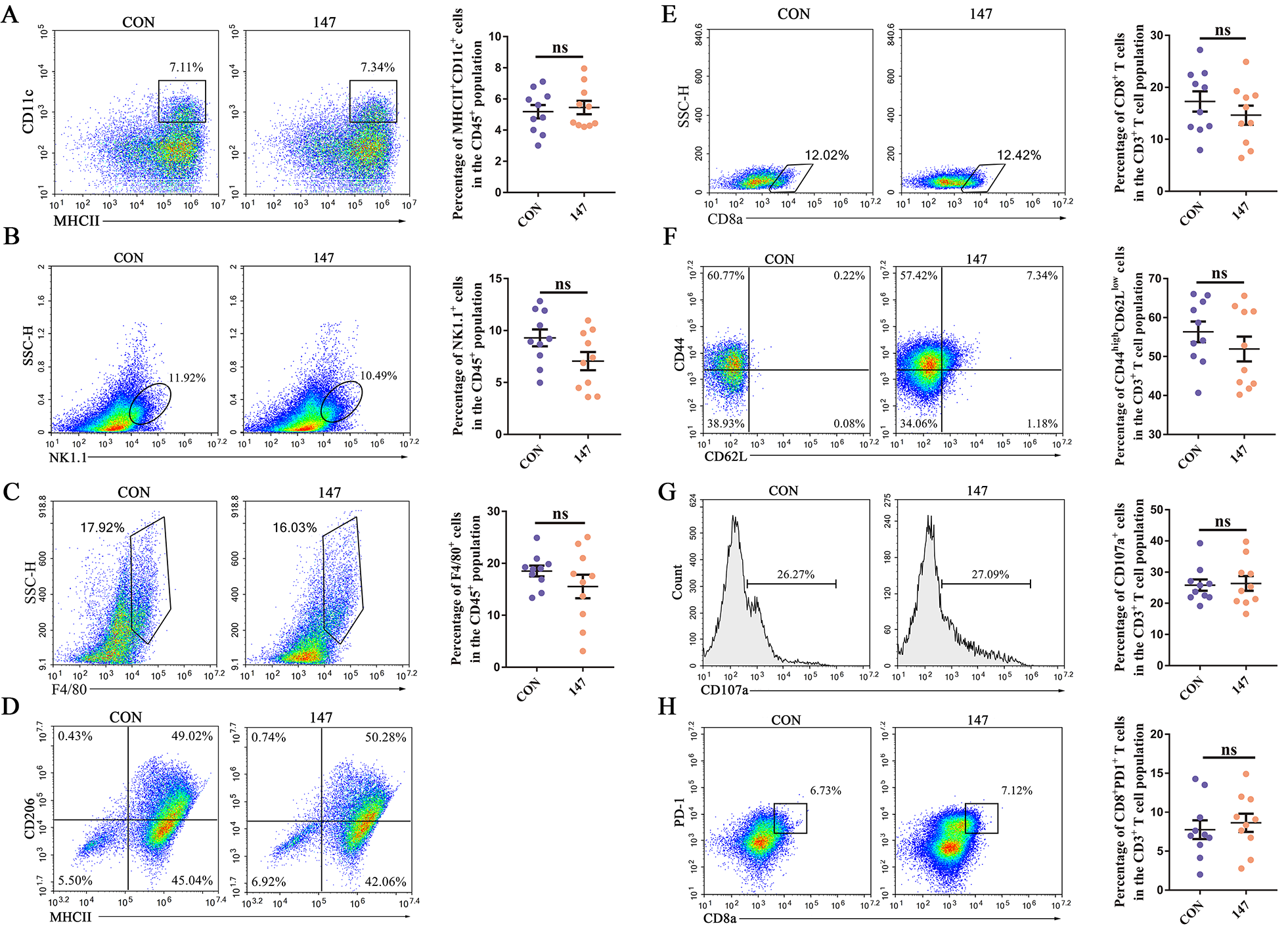


**Supplementary Figure 4.** **The effect of CAR-147 macrophages on tumour immune cell infiltration**. Flow cytometric analysis and quantification of MHCII^+^CD11c^+^ DC cells in the TIL population (n=10) **(A)**, NK1.1^+^ NK cells in the TIL population (n=10) **(B)**, F4/80^+^ tumour-associated macrophages in the TIL population (n=10) **(C),** the expression of CD206 and MHCII in F4/80^+^ tumour-associated macrophages **(D)**, the percentage of CD8^+^ T cells in the CD3^+^ T cell population (n=10) **(E)**, the percentage of CD44^high^CD62L^low^ T cells in the CD3^+^ T cell population **(F)**, the percentage of CD107a^+^ T cells in the CD3^+^ T cell population (n=10) **(G)**, and the percentage of CD8^+^PD-1^+^ T cells in the CD3^+^ T cell population (n=10)**(H)**. All values are expressed as the mean ± SEM. *P < 0.05, **P < 0.01; and ***P < 0.001.


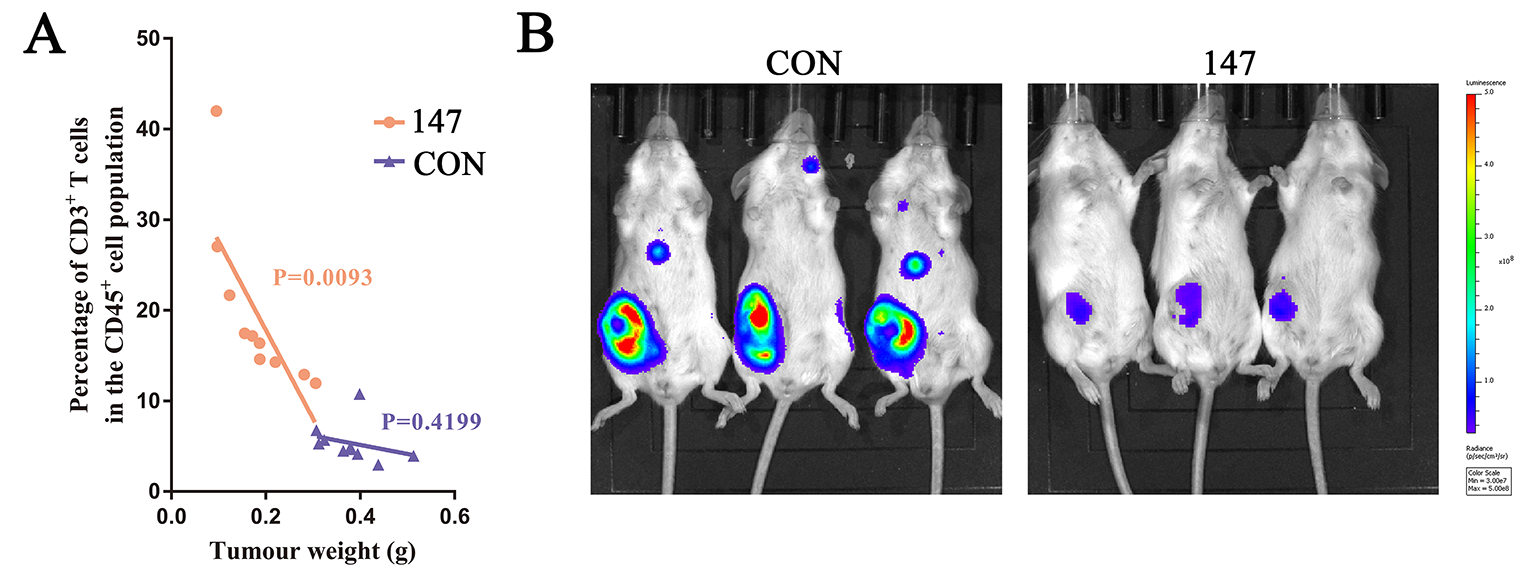


**Supplementary Figure 5. A.** Analysis of the Correlation between tumour weight and the percentage of T cells in tumours from control Raw264.7 cell- or CAR-147 Raw264.7 cell-treated mice. **B.** BLI performed on day 40 to assess tumour metastasis after control or CAR-147 Raw264.7 cell infusion.
